# Effect of Lipopolysaccharides (LPS) and Lipoteichoic Acid (LTA) on the Inflammatory Response in Rumen Epithelial Cells (REC) and the Impact of LPS on Claw Explants

**DOI:** 10.3390/ani11072058

**Published:** 2021-07-09

**Authors:** Nicole Reisinger, Dominik Wendner, Nora Schauerhuber, Elisabeth Mayer

**Affiliations:** BIOMIN Research Center, 3430 Tulln, Austria; dominik.wendner@dsm.com (D.W.); nora.schauerhuber@gmail.com (N.S.); elisabeth.mayer@dsm.com (E.M.)

**Keywords:** rumen epithelial cells, endotoxins, lipopolysaccharides, lipoteichoic acid, claw explants, inflammation

## Abstract

**Simple Summary:**

Endotoxins, often referred to as lipopolysaccharides (LPS), are bacterial toxins and play an essential role in several diseases in ruminants. One of the most common disorders in dairy cows, sub-acute rumen acidosis (SARA), is associated with a substantial increase of ruminal and intestinal endotoxin load. Other potentially harmful substances, e.g., lipoteichoic acid (LTA), derived from the cell wall of Gram-positive bacteria, might play an essential role during SARA as well. Besides the potential local effect of LPS, translocation to the blood can induce a strong immune response in cattle. Furthermore, LPS might reach the claw tissue after translocation. In our study, we used a cell culture model with epithelial cells isolated from rumen tissue to assess the effects of LPS and LTA. Furthermore, we evaluated the effects of LPS on claw tissue with an explant model. LPS and LTA could induce an inflammatory response in rumen epithelial cells. However, the effect of LPS was more substantial and seen at an earlier time point compared to LTA. Furthermore, in claw explants, LPS negatively affected the separation force, an indicator for tissue integrity, which decreased with increasing LPS concentrations. Overall, our data suggest that especially endotoxins can impact local inflammatory response in the rumen. Furthermore, if endotoxins reach the claw tissue, it might affect claw health.

**Abstract:**

Endotoxins play a crucial role in ruminant health due to their deleterious effects on animal health. The study aimed to evaluate whether LPS and LTA can induce an inflammatory response in rumen epithelial cells. For this purpose, epithelial cells isolated from rumen tissue (REC) were stimulated with LPS and LTA for 1, 2, 4, and 24 h. Thereafter, the expression of selected genes of the LPS and LTA pathway and inflammatory response were evaluated. Furthermore, it was assessed whether LPS affects inflammatory response and structural integrity of claw explants. Therefore, claw explants were incubated with LPS for 4 h to assess the expression of selected genes and for 24 h to evaluate tissue integrity via separation force. LPS strongly affected the expression of genes related to inflammation (NFkB, TNF-α, IL1B, IL6, CXCL8, MMP9) in REC. LTA induced a delayed and weaker inflammatory response than LPS. In claw explants, LPS affected tissue integrity, as there was a concentration-dependent decrease of separation force. Incubation time had a strong effect on inflammatory genes in claw explants. Our data suggest that endotoxins can induce a local inflammatory response in the rumen epithelium. Furthermore, translocation of LPS might negatively impact claw health.

## 1. Introduction

Endotoxins, often referred to as lipopolysaccharides (LPS), are part of Gram-negative bacteria’s cell wall. LPS play an essential role in several disorders in ruminants: ruminal acidosis, fatty liver syndrome, claw-related disease, retained placenta, displaced abomasum, etc. [1,2,3,4]. One of the most common disorders in dairy cows, associated with a substantial increase of ruminal and intestinal endotoxin load, is sub-acute rumen acidosis (SARA). Extended periods of depressed ruminal pH characterize SARA conditions, resulting in an imbalance of the microbial population. Consequently, the concentration of LPS can significantly increase in the rumen and gut. Furthermore, translocation of LPS to the blood is possible and is known to cause a strong immune response [2]. Due to the higher consumption of glucose during inflammation, this can result in a drastic drop in milk yield. In a study by Kvidera et al. [5], a decreased milk production of 70% was observed after *i.v.* administration of an LPS bolus. However, the local effects of endotoxins are controversially discussed. In addition to LPS, it is also likely that other compounds derived from bacteria might play an important role in disorders of the gastrointestinal tract, such as SARA. Lipoteichoic acid (LTA), a cell wall component of Gram-positive bacteria, is likely to increase during SARA. However, there is no information about ruminal LTA concentration, its translocation into the blood, and its fate in ruminants.

Overall, it is challenging to assess the direct effects of LPS and LTA on rumen tissue in vivo as most of the challenge models, e.g., high grain challenge, cause an increase of LPS in the rumen and an increase of biogenic amines and other unwanted substances [6]. Only a limited number of studies are available on challenge models in ruminants with an oral bolus of LPS [7,8,9]. Moreover, it needs to be considered that the concentrations of LPS used in these studies might not reflect the substantial increase of ruminal LPS, e.g., during SARA. A grain-based SARA challenge can increase endotoxin concentration in the rumen fluid by 10-fold [6] or even 16-fold [10], which resulted in a ruminal endotoxin concentration of 130,000 and 170,000 EU/mL, respectively. 

Therefore, in vitro test systems are the only possibility to assess the effect of LPS and LTA on the rumen epithelium as a single factor at different time points. However, only a limited number of bovine cell lines are available. For example, calf small intestinal epithelial cells B (CIEB) were used to evaluate the effect of mycotoxins on cell viability [11]. An immortalized bovine ileal epithelial cell line was recently used to evaluate the infectivity and immune responses to bacterial and viral pathogens or ligands [12]. To study rumen epithelial cells, isolation methods from rumen tissue of sheep and cattle are described in the literature [13,14,15], as there is no cell line available. An advantage of primary cells is that they tend to mimic the physiological situation in vivo more accurately. Therefore, the use of isolated rumen epithelial cells (REC) provides a promising tool to assess the effects of LPS and LTA stimulation on the inflammatory response over time. 

In addition to the local effects of endotoxins in the rumen, their translocation and the impact of circulating endotoxins play a vital role in ruminant health. Endotoxin translocation becomes especially important when the liver cannot detoxify endotoxins anymore, e.g., during liver-associated diseases such as hepatic lipidosis [16]. As mycotoxins are described to affect liver health [17], they might affect endotoxin translocation as well. Inflammatory responses, such as cytokine production, caused by circulating endotoxins and other molecules and their effect on claw tissue have already been under discussion for several years [2]. In addition, SARA is associated with higher incidences of lameness or more specific claw disorders, such as discoloration of the hoof, sole hemorrhages, or sole ulceration [18]. It has been shown that endotoxins might play a role as they translocate from the rumen and/or gut to the blood during SARA conditions and potentially negatively affect claw health [3].

However, there is a lack of information on how endotoxins might directly or indirectly harm claw tissue, potentially inducing pathological changes. Only one in vitro study describes the negative effect of LPS on bovine cells isolated from claws. However, only dermal cells and not keratinocytes, one of the essential cell types in the claw, were used in this study [19]. Therefore, a model including keratinocytes and, even better, a model considering the interaction of different tissue parts of the claw (claw wall, lamellar tissue, connective tissue) is not only highly relevant, but still missing. Explant models might provide such an opportunity and were already used to evaluate the effect of endotoxins [20,21,22] and mycotoxins [23] on hoof explants in horses. Therefore, we used explants from the claw, which serve as an important tool to assess the effect of endotoxins on claw health. 

The study aimed to assess the potential direct effect of LPS and LTA on the rumen epithelium and of LPS on claw tissue explants. For the first part, epithelial cells were isolated from rumen tissue and characterized based on immunocytochemistry. Those primary rumen epithelial cells were stimulated with either LPS or LTA, and the expression of genes associated with inflammatory response was evaluated. For the second part, potential effects of LPS on claw tissue were assessed in an in vitro model with bovine claw explants. We evaluated separation force as well as the expression of selected genes associated with inflammation. Overall, this study is the first to provide insight into the direct effects of LPS on claw tissue.

## 2. Materials and Methods

### 2.1. Isolation and Characterization of Primary Rumen Epithelial Cells (REC)

#### 2.1.1. Isolation and Cultivation of Rumen Epithelial Cells

Bovine rumen tissue samples (5 × 5 cm) from dairy cows were obtained post mortem at a local abattoir. No information on breed, age, or cause of culling was available. Tissue samples were washed three times using Dulbecco’s phosphate-buffered saline (DBPS; Gibco Life Technologies, Carlsbad, CA, USA) containing an antibiotic-antimycotic solution (200 Units/mL penicillin, 200 µg/mL of streptomycin, and 0.5 µg/mL amphotericin B; Gibco Life Technologies) to reduce the risk of bacterial or fungal contamination. Tissue samples were kept on ice during transportation to the lab. Before starting dissection, tissue samples were washed once more with DBPS. Further steps were carried out under sterile conditions. 

Papillae were separated from the rumen tissue and cut into small pieces (~5 mm). Cells were enzymatically isolated using trypsin (0.25%; Sigma-Aldrich, St. Louis, MO, USA) and EDTA (0.5 mM; Sigma-Aldrich) in three consecutive extraction steps, 30 min each at 37 °C and 300 rpm. Cell suspension was filtered using a 40 µm cell strainer (Corning Inc., New York, NY, USA), pooled, and centrifuged for 5 min at 400× *g*. Cells were seeded at a density of 5 × 105/well in a coated (Coating Matrix Kit; Gibco Life Technologies) 6-well cell culture plate (Eppendorf, Hamburg, Germany) and incubated at 37 °C and 5% CO_2_. Cultivation medium consisted of Dulbecco’s Modified Eagle Medium (High glucose and pyruvate; Gibco Life Technologies) supplemented with an antibiotic-antimycotic solution (200 Units/mL penicillin, 200 µg/mL of streptomycin, and 0.5 µg/mL amphotericin B; Gibco Life Technologies), 25 mM HEPES (Sigma-Aldrich), 2.5 mM Glutamax (Gibco Life Technologies), 10% fetal bovine serum (Gibco Life Technologies), and 0.04 µg/mL epidermal growth factor (EGF; Gibco Life Technologies). After the second passage, the antibiotic-antimycotic (100 Units/mL penicillin, 100 µg/mL of streptomycin, and 0.25 µg/mL amphotericin B) and EGF (0.01 µg/mL) concentration was reduced. Medium was changed routinely every two days. Cultures were examined daily, observing cell proliferation, morphology, and possible contamination.

#### 2.1.2. Characterization of REC with Immunocytochemistry

Staining of cytokeratin and vimentin was done as previously described by Reisinger et al. [11]. Staining of the tight junction protein occludin was performed with minor modifications: cells were seeded in chamber slides (Eppendorf) at a density of 6 × 10^4^/well. Cells were fixed for 30 min at 4 °C with 4% paraformaldehyde solution (Biotium, Fremont, CA, USA), and a 1% BSA solution (Gibco Life Technologies) was used for blocking. Incubation with primary antibodies was done overnight at 4 °C: rabbit anti-occludin antibody (1:200, ab31721, Abcam) and secondary antibody: goat anti-rabbit IgG H&L FITC antibody (1:500, ab150081, Abcam) for 1 h at room temperature. Fluoroshield mounting medium containing 4′,6-Diamidin-2-phenylindol (DAPI; Abcam, Cambridge, UK) was used for cell nuclei counterstaining and mounting. 

### 2.2. Evaluation of the Effects of LPS and LTA on Gene Expression

#### 2.2.1. Stimulation of REC with LPS and LTA

LPS from Escherichia coli O111:B4 (phenol extraction) and LTA from Streptococcus pyogenes (both Sigma-Aldrich) were weighed (5 mg) in a pyrogen-free glass tube (Charles River Laboratories, Wilmington, MA, USA) and dissolved in 5 mL sterile DPBS. Stock solutions were frozen in pyrogen-free glass tubes (Charles River) at −20 °C. For cell treatment, stock solutions were thawed at room temperature and diluted in culture medium during continuous shaking at 500 rpm. REC (Passage 3–7) were seeded in 12-well plates (Eppendorf) at 2.5 × 10^5^ cells per well. Five independent experiments were performed with cells isolated from two different animals. Cells were incubated at 37 °C and 5% CO_2_. After 24 h, cells were treated with two different LPS and LTA concentrations (1 and 10 µg/mL) in duplicate for 1, 2, 4, and 24 h. Thereafter, supernatant was discarded, cells were washed once with DPBS, and RNAlater Stabilization Solution (Invitrogen, Carlsbad, CA, USA) was added to all wells. Plates were stored at 4 °C overnight and then transferred to −80 °C until RNA extraction. 

#### 2.2.2. RNA Extraction and Gene Expression Analysis of REC

Total RNA was isolated from cells using the RNeasy Mini Kit (Qiagen GmbH, Hilden, Germany): REC were lysed and separated from the culture surface of the 12-well plates, and total RNA was extracted and purified. Isolated RNA samples were sent on dry ice to Qiagen for further analysis. RNA concentration was measured via a Nanodrop spectrophotometer (Thermo Fisher Scientific, MA, USA) and RNA quality was assessed using the RNA TapeScreen (Agilent, Santa Clara, CA, USA). For cDNA synthesis, the RT^2^ First Strand Kit (Qiagen) was used, and for RT-qPCR, the RT^2^ Profiler™ Custom Cow PCR Array (CLAB27028) was used. Threshold cycle (Ct) values for all genes were provided by Qiagen and used for data analysis. RNA Integrity Number (RIN) values were all above 7 (7.8–10.0). The 2^ΔΔCt^ method was used for determining the gene expression. The ∆Ct (normalized Ct) value was calculated for each sample by subtracting the Ct value for the target gene from the mean Ct value of the two housekeeping genes. The mean ∆Ct was calculated for each gene and used for statistical evaluation and expressing the fold change (=2^∆∆Ct^ value). Beta-actin (ACTB) and Glyceraldehyde-3-phosphate dehydrogenase (GAPDH) were used as housekeeping genes for normalization. A list of selected genes and references is provided in Supplementary Table S1.

### 2.3. Effect of LPS on Separation Force and Gene Expression of Bovine Claw Explants

#### 2.3.1. Preparation of Bovine Claw Explants with LPS

Bovine claws were obtained from bulls, post mortem, at a local abattoir. No information on breed, age, or cause of culling was available. Claws were transported within one hour to the lab. Tissue was macroscopically checked for diseases, which might affect tissue integrity. Preparation was done as described previously by Reisinger et al. [22] for equine hoof explants.

#### 2.3.2. Metabolic Activity and Separation Force of Bovine Claw Explants

Bovine claw explants (*n* = 12) were stimulated with LPS from Escherichia coli O55:B5 (1, 10, and 100 µg/mL) for 24 h. Viability and separation force of claw explants were measured as described by Reisinger et al. [22] for equine hoof explants. Briefly, claw explants were fixed in a calibrated force transducer (Sautner, BatschWaagen und EDV, Loosdorf, Austria), and the maximum load to failure was measured in Newton [N].

#### 2.3.3. RNA Extraction and Gene Expression Analysis

For gene expression analysis, explants were incubated for 4 h with 1, 5, and 10 µg/mL LPS. Lamellar tissue and connective tissue were separated from the claw wall and placed in RNAlater directly after dissection and after incubation with LPS. Tissue was stored at 4 °C overnight and then transferred to −80 °C until RNA extraction. RNA extraction, measurement of concentration and quality of RNA, cDNA synthesis, RT-qPCR, and data evaluation were done as described above for REC. RIN values were all above 7 (7.3–9.1). Beta-actin (ACTB) and Glyceraldehyde-3-phosphate dehydrogenase (GAPDH) were used as housekeeping genes for normalization. A list of selected genes and references is provided in Supplementary Table S1.

### 2.4. Statistics

Statistical analyses were performed with GraphPad Prism Version 9.0.0. (GraphPad Software, San Diego, CA, USA). For results of gene expression of REC and claw explants, statistical evaluation was done using the 2^ΔΔct^ method. Results are expressed as “fold-regulation” values. The negative control is set to 1, and cut-off values of <−2 or >2 were used to identify relevant gene expression changes. Data were tested for normal distribution with the Shapiro–Wilk test. If data were normally distributed, an ANOVA was performed followed by Bonferroni as a post-hoc test for the results of the REC stimulated with LPS and LTA. Dunn’s test was used as a post-hoc test for the results of the claw explants stimulated with LPS. A Kruskal–Wallis test was used as a non-parametric test if data were not normally distributed. Differences were considered statistically significant when the *p*-value was <0.05. Principal component analysis (PCA) was performed with the free web tool MetaboAnalyst 4.0 [24]. Software R studio (Version 3.6.3.: Boston, MA, USA) was used to generate heat maps.

## 3. Results

### 3.1. Isolation and Characterization of Primary Rumen Epithelial Cells

**Figure 1:**
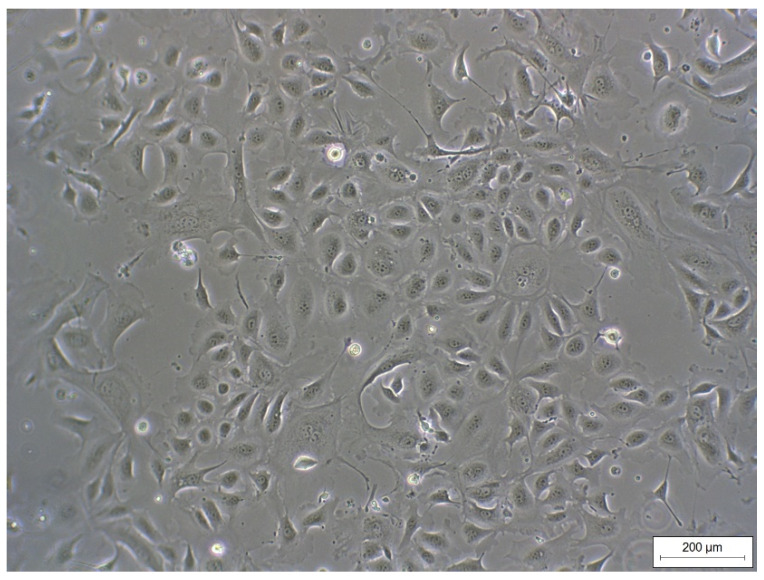
Monolayer of REC (passage 3) isolated from rumen epithelial tissue of dairy cows showing a typical epithelial cobblestone morphology.

REC formed a cell monolayer and showed typical epithelial cobblestone morphology (Figure 1). Morphology did not change up to passage 7 (highest passage used for experiments).

**Figure 2:**
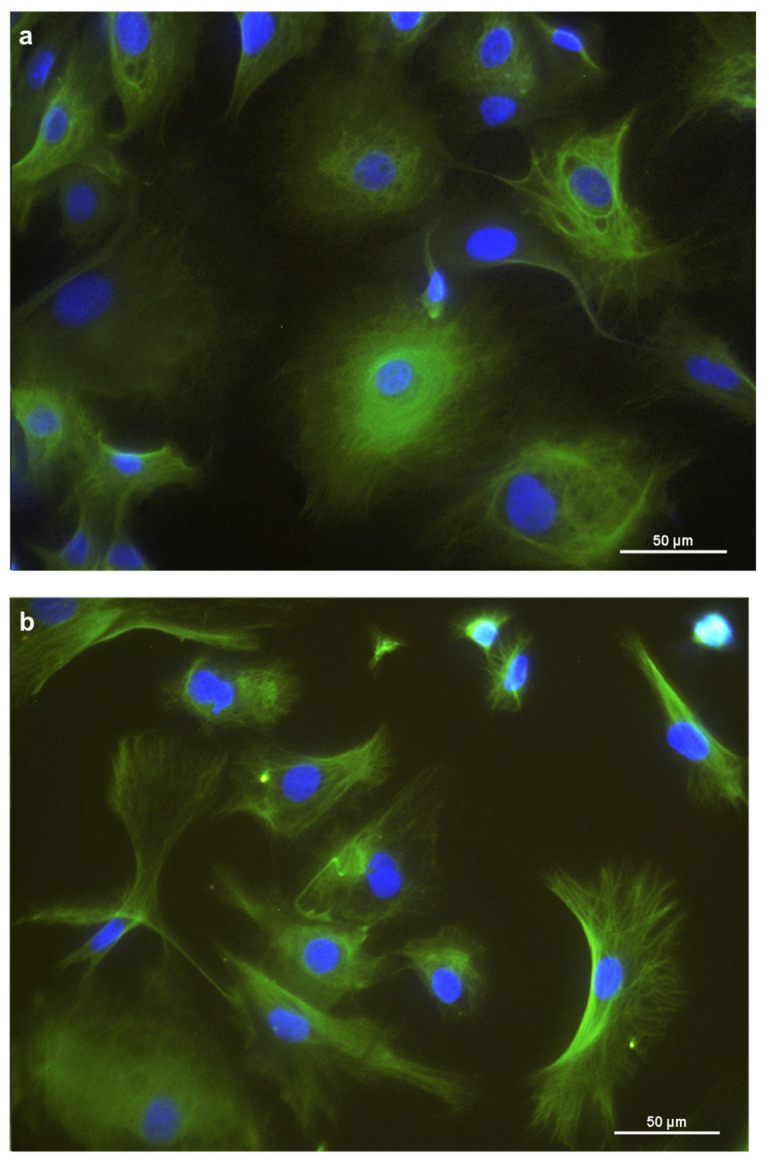

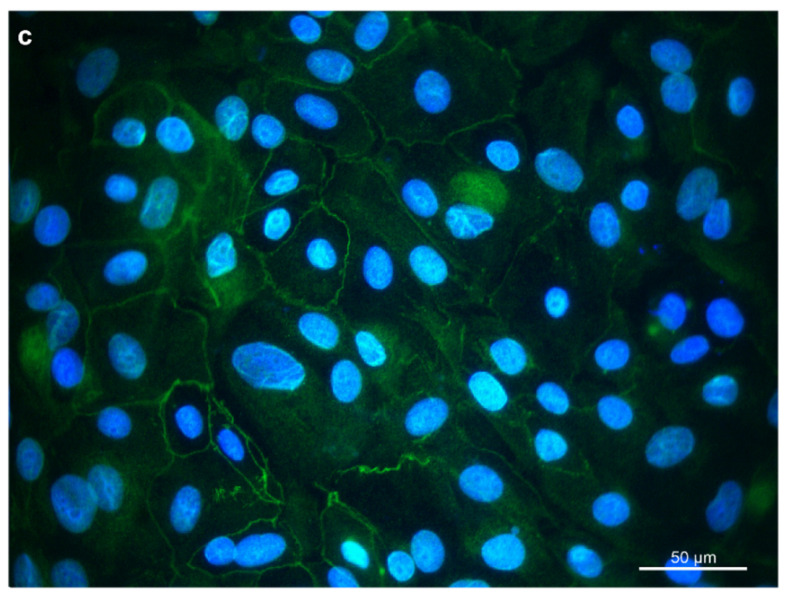
Immunostaining of REC in chamber slides with (**a**) cytokeratin as an epithelial cell marker, (**b**) vimentin as a mesenchymal marker, and (**c**) occludin, a tight junction protein. DAPI was used as a cell nuclei counterstain.

For further characterization, immunocytochemistry was employed. Cytokeratins were expressed in the cytoplasm of the REC (Figure 2a). Vimentin was expressed, forming a filamentous network throughout the cytoplasm. (Figure 2b). Furthermore, REC also expressed the tight junction protein occludin at the cells’ border (Figure 2c). Unspecific background staining in the cytoplasm was seen with occludin as well.

### 3.2. Effects of LPS and LTA on Gene Expression in Primary Rumen Epithelial Cells

**Figure 3:**
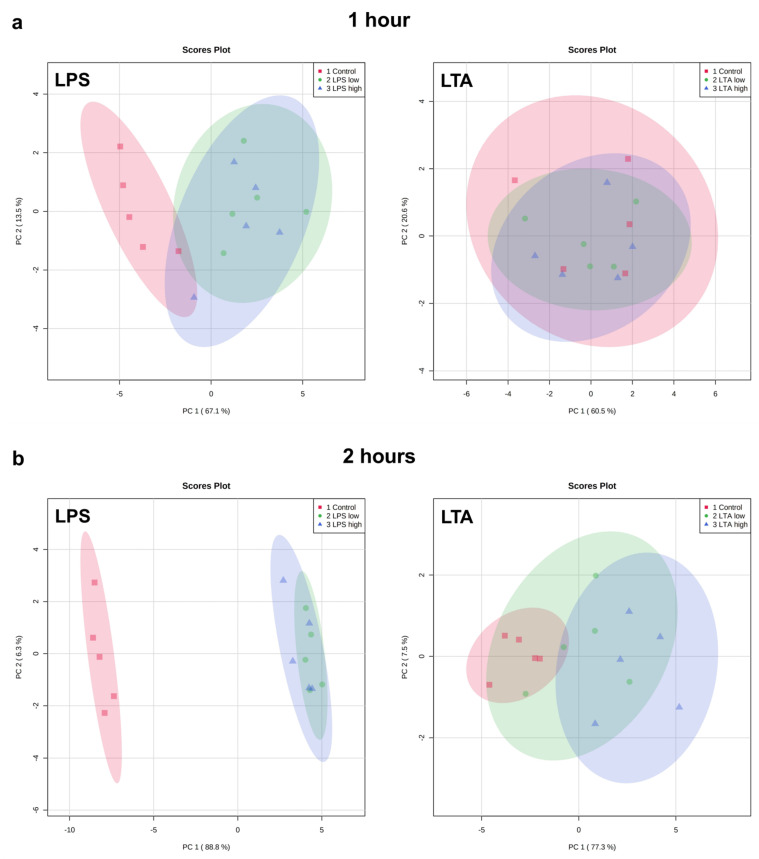

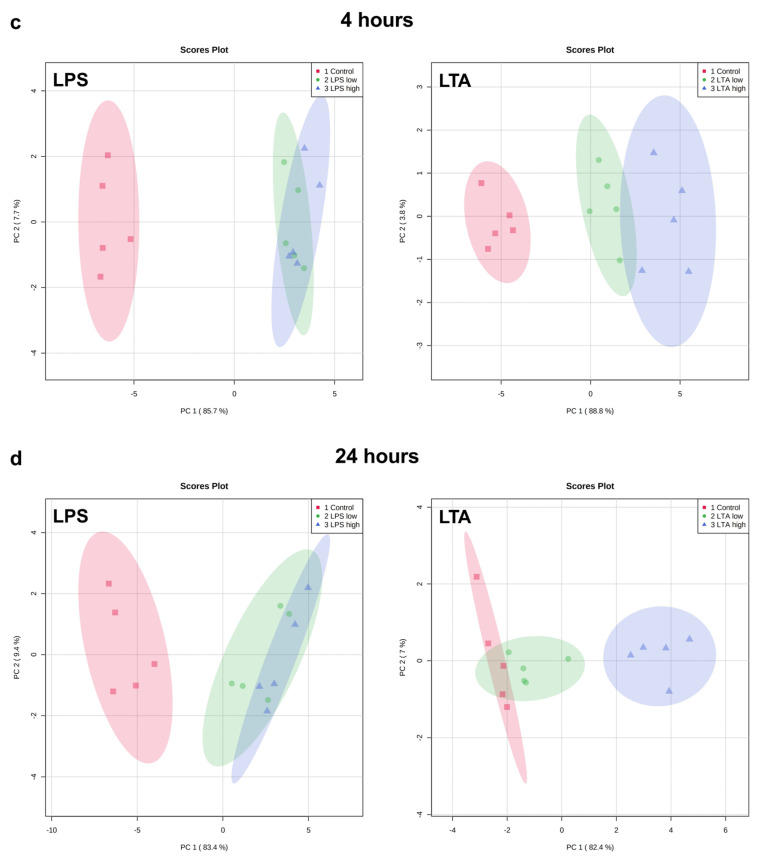
PCA plots of the expression of all measured genes of REC treated with medium only (control), LPS low (1 µg/mL), LPS high (10 µg/mL), LTA low (1 µg/mL), and LTA high (10 µg/mL) for 1 (**a**), 2 (**b**), 4 (**c**), and 24 (**d**) h. *n* = 5 independent experiments.

Exploratory analysis (principal component analysis (PCA) was performed on the expression of all measured genes (Figure 3). The first principal component can explain the major part of the variation in the results. 

Both LPS groups, low (1 µg/mL) and high (10 µg/mL), exhibited a markedly distinct pattern compared to the control group after 1, 2, 4, and 24 h of incubation. The effects were independent of the LPS concentration. While after 1 h, both LTA concentrations did not affect the gene expression profile, after 2, 4, and 24 h, both LTA groups, low (1 µg/mL) and high (10 µg/mL), exhibited a markedly distinct pattern compared to the control group. LTA high showed a different pattern than LTA low at 2, 4, and 24 h. The overall pattern changed in a time-dependent manner when REC were stimulated with LPS as well as LTA.

There was no effect of any treatment on the expression of TLR2, TLR4, MyD88, and TRAF6 (*p* > 0.05; Table 1 and Table 2). The expression of NFKB1 was increased after 2 h, 4 h, and 24 h by both LPS low and LPS high (*p* < 0.05; Table 1). After 1 h of incubation, the expression of TNF-α was only upregulated in the LPS low group, while both LPS concentrations increased the expression of TNF-α after 2 h, 4 h, and 24 h (*p* < 0.05; Table 1, Figure 3).

**Figure 4:**
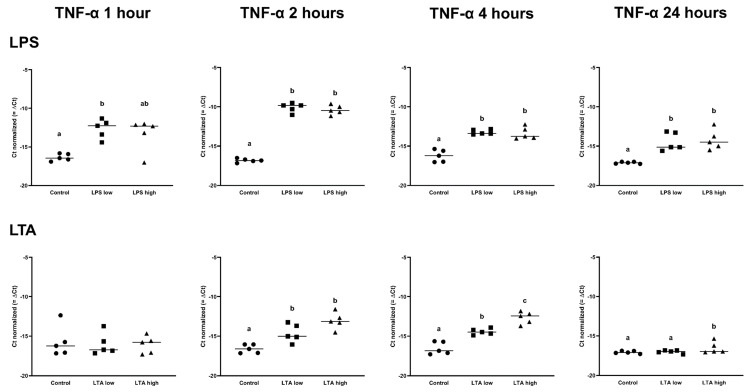
Delta Ct values of TNF-α of REC treated for 1, 2, 4, and 24 h with 0 µg/mL (Control), 1 µg/mL LPS (LPS low), 10 µg/mL (LPS high), 1 µg/mL LTA (LTA low), and 10 µg/mL LTA (LTA high). ^a,b,c^ Superscripts indicate significant differences within 1, 2, 4, and 24 h: *p* < 0.05. *n* = 5 independent experiments.

In contrast, the LTA low and high groups both only upregulated the expression of TNF-α after 2 and 4 h (*p* < 0.05), while at 24 h, only LTA high upregulated the expression of TNF-α (*p* < 0.05) (Figure 4, Table 2).

**Figure 5:**
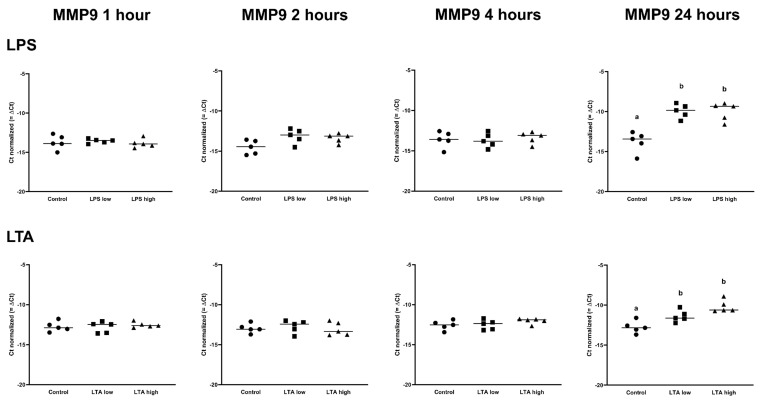
Delta Ct values of MMP9 of REC treated for 1, 2, 4, and 24 h with 0 µg/mL (Control), 1 µg/mL LPS (LPS low), 10 µg/mL (LPS high), 1 µg/mL LTA (LTA low), and 10 µg/mL LTA (LTA high). *n* = 5 independent experiments. ^a,b^ Superscripts indicate significant differences within 1, 2, 4, and 24 h: *p* < 0.05.

IL1B was upregulated in both LPS low and high, after 1 h, 2 h, and 4 h (*p* < 0.05), while IL1B was only upregulated in the LPS high group at 24 h (*p* < 0.05; Table 1). LTA low and LTA high both increased the expression of IL1B after 2 h of incubation. In contrast, only LTA high increased the expression of IL1B after 4 h and 24 h (*p* < 0.05; Table 2). IL6 expression was increased by both LPS low and LPS high after 2 h, 4 h, and 24 h (*p* < 0.05; Table 1), while the expression of IL6 was only increased in the LTA high group at 2 h (*p* < 0.05; Table 2). CXCL8 was upregulated by LPS low and LPS high after 1 h, 2 h, 4 h, and 24 h of incubation (*p* < 0.05; Table 1), while CXCL8 was only upregulated by the LTA high group at 2 and 24 h (*p* < 0.05; Table 2). There was no effect of any treatment on MMP2 expression. MMP9 was only upregulated after 24 h by LPS low and high as well as LTA low and high (*p* < 0.05; Table 1 and Table 2, Figure 5.

### 3.3. Effects of LPS on Tissue Integrity and Gene Expression of Bovine Claw Explants

**Figure 6:**
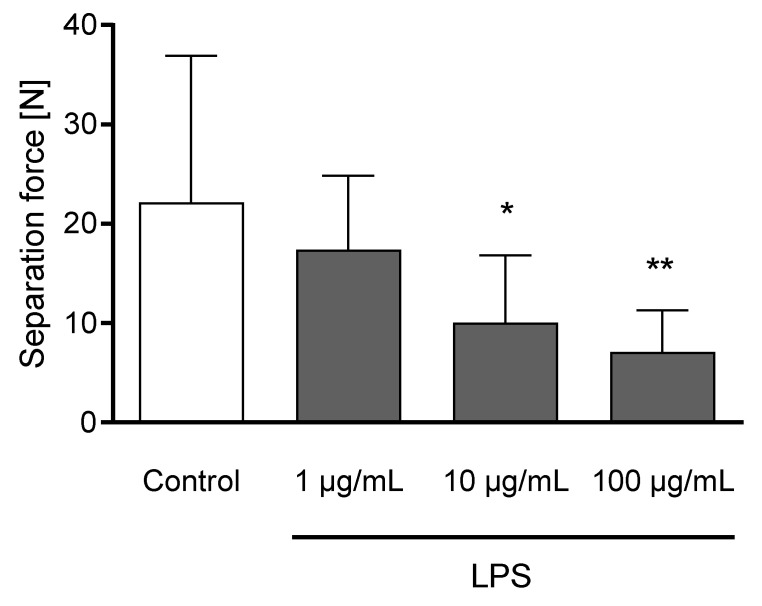
Separation force [N] of untreated explants (Control) and explants incubated with 1, 10, and 100 µg/mL LPS) for 24 h. *n* = 12 explants. Error bars present standard deviation. * Asterisks indicate significant differences: * *p* < 0.05, ** *p* < 0.01.

The incubation of claw explants with 10 and 100 µg/mL LPS for 24 h led to a decreased separation force (*p* < 0.05; Figure 6). Explants remained metabolically active after 24 h of incubation (Figure S1).

**Figure 7:**
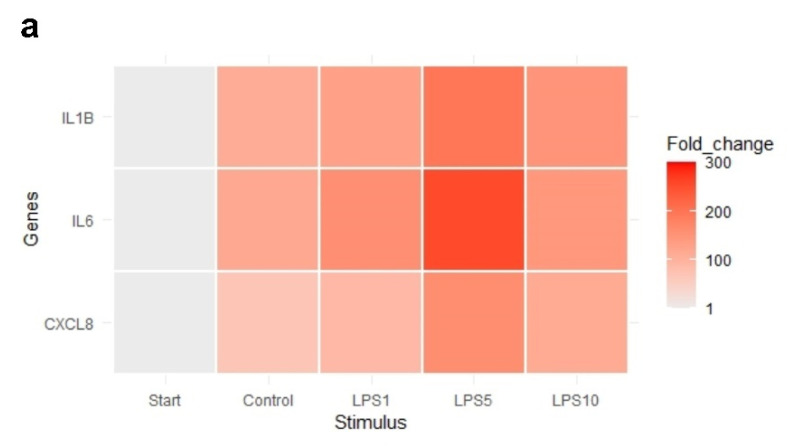

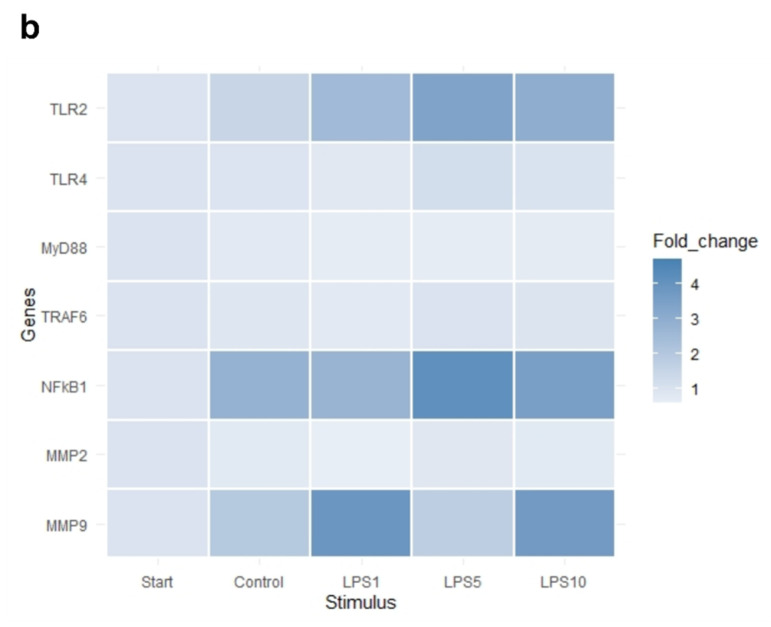
Heat map of the expression of (**a**) highly affected and (**b**) less affected genes of control explants before incubation (Start), untreated explants (Control) after 4 h of incubation, and explants incubated with 1, 5, and 10 µg/mL LPS (LPS1, LPS5, LPS10) for 4 h. *n* = 5 animals.

The heat map in Figure 7 indicates that an incubation time of 4 h strongly affected the expression of IL1B, IL6, and CXCL8. Exploratory analysis (PCA) was performed on the expression of all measured genes (Figure S2). The first principal component can explain the major part of the variation in the results. Control explants at 0 h exhibited a markedly distinct pattern compared to the control explants after 4 h.

There was no effect of any treatment on expression of TLR4, MyD88, and TRAF6 (*p* > 0.05; Table 3). Expression of TLR2 was upregulated after 4 h of incubation when explants were incubated with 1, 5, or 10 µg/mL LPS (*p* < 0.05; Table 3). NFKB1, IL1B, and CXCL8 expression was upregulated in control explants (*p* < 0.05) and explants incubated with 1, 5, or 10 µg/mL LPS (*p* < 0.05) after 4 h of incubation compared to control explants at 0 h (Table 3). TNF-α expression was upregulated in control explants (*p* < 0.05) and explants incubated with 5 or 10 µg/mL LPS (*p* < 0.05) after 4 h of incubation compared to control explants at 0 h (Table 3). There was no difference in the expression of genes comparing control explants after 4 h of incubation to explants incubated for 4 h with 1, 5, or 10 µg/mL (*p* > 0.05).

## 4. Discussion

Endotoxins play a crucial role in ruminants due to their deleterious effects on animal health. We, therefore, used an in vitro model with rumen epithelial cells to elaborate the direct effects of LPS and LTA on the rumen epithelium. This model provides an important tool to mimic the increased endotoxin load after disturbances of the gastrointestinal health. Furthermore, to evaluate the effects of circulating endotoxins on claw health, an in vitro explant model was used. 

In our study, we established a rumen epithelial cell model with cells isolated from rumen tissue, as no commercial cell line is currently available. Protocols to isolate rumen epithelial cells are already described in different ruminant species, e.g., cattle [15], sheep [25], and goat [26]. Bacterial contamination and growth of fibroblasts are common problems when working with primary cells. A proliferating REC culture without contamination could be achieved after slight adaptions of the protocols. Another essential part is the identification of the epithelial origin of REC via immunocytochemistry. We confirmed that REC were epithelial cells with functional properties, e.g., expressing tight junction proteins. We used cytokeratin as an epithelial cell marker, and vimentin as a potential marker of fibroblasts. Both markers were used in other studies to characterize rumen [27] and intestinal cells [11]. REC were stained positive for cytokeratin; however, they were stained positive for vimentin as well. In some studies, vimentin is considered as a typical marker for non-epithelial cells, e.g., fibroblasts.

Interestingly, vimentin expression was already reported in primary rumen epithelial cells [27], a calf intestinal epithelial cell line [11], and cells isolated from the calf intestine [28]. Therefore, vimentin is not an appropriate marker for fibroblasts in cells derived from rumen tissue. Thus, the identification of fibroblasts to ensure the purity of primary cells is still challenging. Although positive expression of cytokeratin suggests that REC are of epithelial origin, another marker was included for identification via immunocytochemistry. Cells were stained for occludin, a tight junction protein characteristically expressed by barrier-forming cells. This tight junction protein is not expressed by cells originating from connective tissues, e.g., fibroblasts. Notably, occludin was used together with cytokeratin in another study to identify epithelial origin in sheep rumen epithelial cells [27].

To study the direct effects of LPS and LTA on rumen epithelium, we used REC and evaluated the expression of genes related to the inflammatory response. Overall, our data showed a strong effect of LPS on the expression of several genes related to inflammation. 

Of note, we did not see any effect of LPS on the expression of TLR4. This result is supported by previous findings of our research group with other cell types, namely mouse macrophages, after LPS stimulation for 4 and 24 h [29]. We hypothesize that due to the strong upregulation of proinflammatory cytokines already after 1 h of LPS stimulation, the lack of effect on TLR4, MyD88, and TRAF6 might be a consequence of a negative feedback loop as described by Lichte et al. [30]. Nonetheless, our results are in contrast to the studies by Kent-Dennis et al. [13,14], which showed an upregulation of TLR4 after LPS stimulation for 6 h. Still, the authors emphasized that TLR4 upregulation is strongly dependent on the time point. Notably, studies evaluating the response in ruminants challenged by high grain diets to induce SARA did not see an upregulation of the TLR4 expression [31,32] as well. This might reflect that TLR4 upregulation is highly dependent on the time point, host, and previous exposure to LPS. A decrease of TLR4 in dairy cows might be a mechanism to adapt to LPS and avoid strong inflammatory activation [33]. It is suggested that the downregulation of TLR4 expression in the ruminal epithelium after calving is part of an adaptation mechanism to high-concentrate diets [34]. In contrast, a study by Chen et al. [35] described that the decrease of TLR4 led to a higher susceptibility of steers to acidosis. Animals showing a higher expression were more resistant to acidosis. Therefore, the role of TLR4 in the rumen epithelium seems to be complex and still needs to be elucidated in more detail.

Nevertheless, we could see a strong upregulation of inflammatory genes TNF-α, IL1B, IL6, and CXCL8 in our study. These results reflect what is seen in other studies with rumen epithelial cells [13,14,36]. Especially for TNF-α, a time-dependent effect could be observed in our study, peaking at 2 h. Again, we want to highlight that based on our results and results of other research groups, the time point after LPS exposure is a critical issue and needs to be considered when performing studies with LPS. Furthermore, it needs to be considered that no information on breed, age, or cause of culling of the animals was available. This might have affected results, especially in regards to inflammatory response. In addition to the common proinflammatory genes, we were interested in evaluating the effect of LPS on two different matrix metalloproteinases (MMPs), MMP2 and MMP9. MMPs are endopeptidases, which play an essential role in tissue remodeling, thereby regulating the homeostasis of the extracellular matrix. Several studies suggest that MMP2 and MMP9 play an essential role in intestinal inflammation [37,38] and were described to increase gut permeability [39,40]. This increase might be a consequence of the degradation of tight junction proteins, which can be caused by MMP9. Therefore, MMPs might also play an essential role in rumen and gut barrier failure during acidosis. Although MMP9 is expressed in the rumen tissue [41], this is the first study evaluating the effect of LPS on MMP9 expression in rumen epithelial cells. We could observe an upregulation of MMP9 after 24 h. However, MMP2 was not affected. The increased expression of MMP9 after LPS stimulation is consistent with other in vitro studies using different cell types [42,43,44,45]. For example, the upregulation of MMP9, but not MMP2, has been described in a study with LPS stimulation using fibroblast [44]. However, only a limited number of studies describe the effect of, e.g., SARA challenge models, on the expression of MMP2 and MMP9 in the rumen epithelium. For instance, Dai et al. [46] described an increase in MMP2 and MMP9 expression after high grain feeding. Our data suggest that LPS indirectly affect the barrier due to increased expression of MMP9. Nevertheless, the expression of different tight junction proteins after LPS stimulation needs to be evaluated in further studies.

In addition to LPS, we evaluated the effect of LTA on rumen epithelial cells. In our study, the effect of LTA was less pronounced regarding inflammatory response and delayed compared to LPS. Although the role of LTA in SARA is still not clarified, LTA plays an essential role in mastitis. Therefore, several studies were done with mammary gland cells. A recent study by Wu et al. [47] compared the effect of peptidoglycan, LTA, and LPS on the gene expression of cytokines (IL1B, IL6, CXCL8, and TNF-α) after 24 h. This study supports our data that LTA had a less pronounced effect on the expression of cytokines compared to LPS. However, in this study, it needs to be mentioned that the effects of LPS after 24 h were more substantial than in our study. Similar effects of LPS and LTA on inflammatory response can also be found in other ruminant species such as goats. Bulgari et al. [48] reported a weaker inflammatory response in primary goat mammary epithelial cells after LTA stimulation than LPS. In our study, induction of inflammatory response was delayed and less pronounced when REC were stimulated with LTA compared to LPS. These results are in accordance with results of other studies. A study using inactivated *E. coli* and *S. aureus* to challenge bovine mammary epithelial cells reported a faster inflammatory response to inactivated *E. coli* than *S. aureus* [49]. A recent study by Tsugamia et al. [50] compared the influence of 10 µg/mL LPS and LTA on the inflammatory response of lactating bovine mammary epithelial cells. Several genes related to inflammation, e.g., IL1B and TNF, were more upregulated by LPS compared to LTA. Moreover, a different response can be seen in vivo with intramammary injections of LPS and LTA [51,52]. Furthermore, an intramammary challenge of LPS is also accompanied by a higher degree of pain and discomfort compared to LTA in cows [53]. 

LPS concentrations used in our study to stimulate REC reflect concentrations observed during experimentally induced acidosis by high grain diet challenge in vivo. As there is no information available regarding ruminal concentration, LTA concentration was chosen based on the LPS concentration. Interestingly, we could not see a concentration-dependent increase in the expression of genes after LPS stimulation. These data are in accordance with other studies, which observed that LPS is capable of inducing a strong inflammatory response regardless of the used LPS concentrations [13]. Overall, it needs to be mentioned that not only the concentration of LPS or LTA can increase in the rumen. Therefore, the interaction of those two stimuli and other toxins, e.g., mycotoxins and biogenic amines, needs to be considered for further studies. These multiple factors might even result in a more substantial effect on the inflammatory response. 

Altogether, our data show that endotoxins can induce an inflammatory response and might alter tight junction proteins via an increase of MMP9 expression in the rumen. LTA induced a weaker inflammatory response with a time delay compared to LPS in rumen epithelial cells.

Besides potential local effects of endotoxins on the rumen epithelium, a key aspect is the translocation of LPS, and potentially LTA, into the bloodstream due to an impaired rumen or gut barrier. For several conditions, including transition period, an increase of plasma endotoxins in the blood is described [1,2,3,4]. Impaired liver health can play an important role as well, as it can lead to an increased endotoxin concentration or delayed endotoxin clearance [16]. Endotoxins are often discussed to play a role in the ethology of claw diseases, e.g., claw wall horn disruption [54]. We, therefore, were interested in evaluating the effect of LPS on claw tissue, as there are hardly any studies on the effects of endotoxins on claw tissue. The reasons for that could be explained by a lack of appropriate models to evaluate the pathology after LPS exposure. For our study, we used an explant model already described in horses [20,21]. This model mimics a sudden increase of endotoxins in the bloodstream reaching the claw tissue, but cannot reflect the effect of chronic exposure to endotoxins over days or even weeks. Our data show that endotoxins strongly affected the tissue integrity of the claw explants after 24 h. This is in accordance with studies in horses, where similar concentrations led to the separation of explants after LPS exposure for 24 h [21]. Furthermore, a study with Holstein bulls showed that the infusion of 20 µg/mL LPS led to degenerative changes in the papillae and laminae [55]. In addition to the structural changes induced by LPS, we evaluated the inflammatory response in claw explants. However, there was only an upregulation of TLR2 expression of explants incubated with LPS compared to control explants at 4 h. The lack of effect of LPS on other genes can be explained by the strong effect of incubation time on the expression of NFKB1, IL1B, IL6, CXCL8, and TNF-α. For IL1B and IL6, a 111-fold and 117-fold increase was observed when comparing explants right after dissection and explants after incubation for 4 h without adding LPS. For further studies, the strong effect of incubation times needs to be considered. Either shorter incubations times are needed, or the tissue needs to adapt to the culture conditions before stimulating explants with LPS. However, a longer adaption time might be critical as the incubation time is limited by decreased tissue viability over time [21]. This might be overcome by adjusting the medium components and thickness of the tissue to ensure nutrient supply. LPS concentrations used in our study to stimulate claw explants are comparable to studies using either fibroblast or keratinocytes. Both are essential cell types of the claw hoof tissue. Tian et al. [19] showed an increase in cytokine concentration of IL1B and TNF-α in the supernatant when incubating dermal claw cells with 10 µg/mL for 24 h. Furthermore, stimulation of equine keratinocytes, with 5 µg/mL LPS for 4 and 24 h, significantly increased the expression of IL1B, IL6, and CXCL8 [56]. However, it always needs to be considered that claw-related diseases are multifactorial, and certain other factors, e.g., exotoxins, biogenic amines, and mycotoxins, need to be considered.

To conclude, the claw explant model is challenging in regards to cultivation time and conditions. Therefore, optimization needs to be done to use this model for future studies. 3D cell culture models, combining the essential cell types present in the claw, keratinocytes, and fibroblast, might provide an alternative evaluating the prolonged effects of toxins on claw tissue in the future.

## 5. Conclusions

Endotoxins play an essential role in ruminants, as the endotoxin amount can drastically increase in the gastrointestinal tract during SARA. Our data suggest that endotoxins have the potential to induce a local inflammatory response in the rumen epithelium. As endotoxins cross the rumen and gut barrier, e.g., during SARA, as a consequence, other tissues might be affected by endotoxins as well. The claw explant model showed that endotoxins negatively affected tissue integrity. Overall, the impact of endotoxins on health and welfare in ruminants should not be underestimated. In vitro models either with isolated cells or using explants can help and support in vivo research to elucidate the role of endotoxin in the etiology of several disorders in ruminants.

## Figures and Tables

**Table 1:** Expression of measured genes of REC treated with medium only (Control), LPS low (1 µg/mL), and LPS high (10 µg/mL) for 1, 2, 4, and 24 h. *n* = 5 independent experiments. Values are fold changes expressed relative to the untreated cells (Control). Control was set to 1.0.

Genes	1 h			2 h			4 h			24 h		
	Control	LPS Low	LPS High	Control	LPS Low	LPS High	Control	LPS Low	LPS High	Control	LPS Low	LPS High
TLR2	1.00	−1.27	−1.23	1.00	1.26	1.51	1.00	1.45	1.32	1.00	1.36	1.24
TLR4	1.00	−1.34	−1.20	1.00	−1.05	−1.18	1.00	1.43	1.17	1.00	1.26	1.07
MyD88	1.00	1.08	1.02	1.00	1.16	−1.10	1.00	1.05	−1.03	1.00	1.09	1.07
TRAF6	1.00	−1.20	−1.24	1.00	1.01	1.02	1.00	1.06	1.03	1.00	1.01	1.02
NFKB1	1.00	1.09	1.02	1.00 ^a^	2.39 ^b^	2.13 ^b^	1.00 ^a^	2.34 ^b^	2.23 ^b^	1.00 ^a^	2.09 ^b^	2.04 ^b^
TNF-α	1.00 ^a^	12.92 ^b^	8.04 ^ab^	1.00 ^a^	106.03 ^b^	87.13 ^b^	1.00 ^a^	8.11 ^b^	7.33 ^b^	1.00 ^a^	6.33 ^b^	7.63 ^b^
IL1B	1.00	1.90	−1.05	1.00 ^a^	43.48 ^b^	46.93 ^b^	1.00 ^a^	96.98 ^b^	113.43 ^b^	1.00 ^a^	16.75 ^ab^	38.03 ^b^
IL6	1.00	2.31	2.61	1.00 ^a^	26.55 ^b^	22.71 ^b^	1.00 ^a^	10.73 ^b^	10.14 ^b^	1.00 ^a^	6.75 ^b^	6.97 ^b^
CXCL8	1.00 ^a^	24.87 ^b^	21.99 ^b^	1.00 ^a^	187.90 ^b^	158.33 ^b^	1.00 ^a^	29.47 ^b^	37.00 ^b^	1.00 ^a^	34.49 ^b^	42.50 ^b^
MMP2	1.00	1.15	1.15	1.00	1.05	−1.00	1.00	1.04	1.17	1.00	1.02	−1.00
MMP9	1.00	1.10	−1.11	1.00	2.59	2.20	1.00	1.45	1.32	1.00 ^a^	14.32 ^b^	13.77 ^b^

^a,b^ Means without a common superscript indicate significant differences (*p* < 0.05), when compared within a respective timepoint.

**Table 2:** Expression of measured genes of REC treated with medium only (Control), LTA low (1 µg/mL), and LTA high (10 µg/mL) for 1, 2, 4, and 24 h. *n* = 5 independent experiments. Values are the fold changes expressed relative to the untreated cells (Control). Control was set to 1.0.

Genes	1 h			2 h			4 h			24 h		
	Control	LTA Low	LTA High	Control	LTA Low	LTA High	Control	LTA Low	LTA High	Control	LTA Low	LTA High
TLR2	1.00	1.34	−1.23	1.00	1.01	−1.08	1.00	1.26	1.13	1.00	1.20	1.75
TLR4	1.00	−1.23	−1.10	1.00	−1.03	−1.17	1.00	1.01	−1.02	1.00	1.01	1.17
MyD88	1.00	1.06	1.03	1.00	1.05	1.05	1.00	1.01	−1.01	1.00	−1.05	1.03
TRAF6	1.00	−1.11	−1.05	1.00	−1.03	−1.01	1.00	−1.00	1.01	1.00	1.00	−1.01
NFKB1	1.00	1.01	1.01	1.00	1.57	2.06	1.00	1.15	1.27	1.00	1.21	1.52
TNF-α	1.00	−1.24	−1.28	1.00 ^a^	4.23 ^b^	14.45 ^b^	1.00 ^a^	3.93 ^b^	11.73 ^c^	1.00 ^a^	1.37 ^a^	2.86 ^b^
IL1B	1.00	−1.02	−1.05	1.00 ^a^	19.56 ^b^	60.72 ^b^	1.00 ^a^	1.36 ^ab^	2.95 ^b^	1.00 ^a^	1.28 ^a^	20.8 ^b^
IL6	1.00	1.44	1.61	1.00 ^a^	2.72 ^a^	5.53 ^b^	1.00	1.01	−1.02	1.00	1.05	1.47
CXCL8	1.00	1.14	1.34	1.00 ^a^	12.67 ^a^	54.67 ^b^	1.00	1.26	1.13	1.00 ^a^	1.80 ^a^	7.07 ^b^
MMP2	1.00	1.13	1.14	1.00	−1.02	1.00	1.00	−1.00	1.01	1.00	1.15	1.11
MMP9	1.00	−1.06	1.16	1.00	1.05	1.45	1.00	1.17	−1.06	1.00 ^a^	2.57 ^b^	6.01 ^b^

^a,b,c^ Means without a common superscript indicate significant differences (*p* < 0.05), when compared within a respective timepoint.

**Table 3:** Expression of measured genes of control explants before incubation, control explants after 4 h of incubation, and explants incubated for 4 h with 1, 5, and 10 µg/mL LPS. Values are fold changes expressed relative to the control explants before incubation (Control, 0 h). *n* = 5 animals.

Genes	Start 0 h	Control 4 h	1 µg/mL LPS 4 h	5 µg/mL LPS 4 h	10 µg/mL LPS 4 h
TLR2	1.00 ^a^	1.51 ^a^	2.52 ^b^	3.39 ^b^	3.0 ^b^
TLR4	1.00	−1.05	−1.25	1.17	1.03
MyD88	1.00	−1.27	−1.47	−1.45	−1.39
TRAF6	1.00	−1.14	−1.28	1.00	−1.05
NFKB1	1.00 ^a^	2.83 ^b^	2.73 ^b^	4.13 ^b^	3.55 ^b^
TNF-α	1.00 ^a^	2.79 ^b^	1.31^a^	4.70 ^b^	4.65 ^b^
IL1B	1.00 ^a^	110.92 ^b^	130.86 ^b^	197.04 ^b^	150.52 ^b^
IL6	1.00 ^a^	117.49 ^b^	159.27 ^b^	255.75 ^b^	144.44 ^b^
CXCL8	1.00 ^a^	68.71 ^b^	91.30 ^b^	162.57 ^b^	115.19 ^b^
MMP2	1.00	−1.25	−1.64	−1.19	−1.25
MMP9	1.00	1.97	3.89	1.77	3.72

^a,b^ Means without a common superscript indicate significant differences (*p* < 0.05).

## Data Availability

Not applicable.

## Supplementary Materials

The following are available online at https://www.mdpi.com/article/10.3390/ani11072058/s1, Table S1: Selected genes and references analyzed in REC and claw explants. Figure S1. Absorbance values at 450 nm measured with the WST-1 assay of control explants after 24 h of incubation with medium. Error bars display the standard deviation. Figure S2. PCA plots of the expression of all measured genes of explants before incubation (Start), untreated explants (Control) after 4 h of incubation, and ex-plants incubated with 1, 5, and 10 µg/mL LPS (LPS1, LPS5, LPS10) for 4 h. *n* = 5 animals.
