# Supplementary material for: Effect of Lipopolysaccharides (LPS) and Lipoteichoic Acid (LTA) on the Inflammatory Response in Rumen Epithelial Cells (REC) and the Impact of LPS on Claw Explants
Source: Animals (Basel). 2021 Jul 9;11(7):2058. doi: 10.3390/ani11072058 (PMC8300308; doi:10.3390/ani11072058)
Supplement: Supplementary file 1 [file animals-11-02058-s001.zip › animals-1248896-supplementary.pdf]

Supplementary: Effect of lipopolysaccharides (LPS) and lipoteichoic acid (LTA) on inflammatory response in rumen epithelial cells (REC) and the impact of LPS on claw explants

Nicole Reisinger, Dominik Wendner, Nora Schauerhuber, and Elisabeth Mayer

Table S1: Selected genes and references analysed in REC and claw explants.

**Selected genes (LPS pathway, Inflammation)**	**Reference**
CD4 antigen (CD14)	NM_001103225
Interleukin-1 beta (IL-1 beta)	NM_174093, XM_005212737
Matrix metalloproteinase 2 (MMP2)	NM_174745
Matrix metalloproteinase 9 (MMP9)	NM_174744
Myeloid differentiation factor 88 (MyD88)	NM_001014382
Nuclear factor kappa-B (Nfkb)	NM_001076409
Toll-like receptor 2 (TLR2)	NM_174197
Toll-like receptor 4 (TLR4)	NM_174198
Tumor necrosis factor (TNF)	NM_173966, XM_005223596
TNF receptor associated factor (TRAF6)	NM_001034661
**Housekeeping genes**	
Beta actin (ACTB)	NM_173979
Glyceraldehyde-3-phosphate dehydrogenase (GAPDH)	NM_001034034

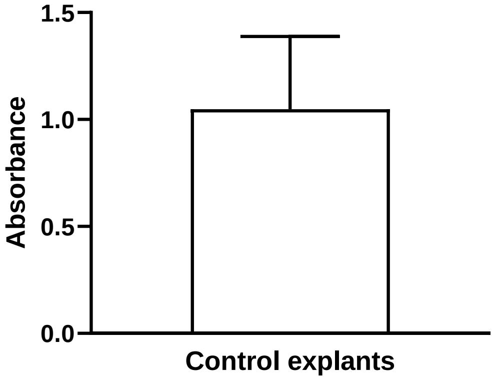

Figure S1. Absorbance values at 450 nm measured with the WST-1 assay of control explants after 24 h incubation with medium. Error bars display standard deviation.

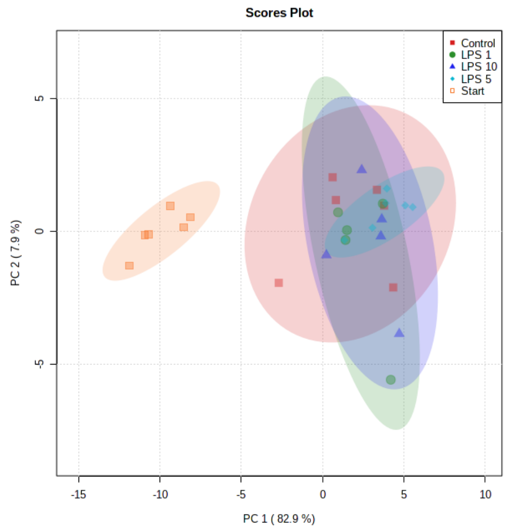

Figure S2. PCA plots of the expression of all measured genes of explants before incubation (Start), untreated explants (Control) after 4 hours incubation, and ex-plants incubated with 1, 5, and 10 µg/mL LPS (LPS1, LPS5, LPS10) for 4 hours. n = 5 animals.
